# Berberine as a Bioactive Alkaloid: Multi-Omics Perspectives on Its Role in Obesity Management

**DOI:** 10.3390/metabo15070467

**Published:** 2025-07-09

**Authors:** Bartłomiej Zieniuk, Magdalena Pawełkowicz

**Affiliations:** 1 Department of Chemistry, Institute of Food Sciences, Warsaw University of Life Sciences-SGGW, 159C Nowoursynowska Str., 02-776 Warsaw, Poland; bartlomiej_zieniuk@sggw.edu.pl; 2 Department of Plant Genetics, Breeding and Biotechnology, Institute of Biology, Warsaw University of Life Sciences-SGGW, 159 Nowoursynowska Str., 02-776 Warsaw, Poland

**Keywords:** alkaloids, metabolomics, multi-omics, obesity management, lipid metabolism, natural products, insuline

## Abstract

Berberine, a bioactive isoquinoline alkaloid derived from medicinal plants such as *Berberis* and *Coptis* species, shows significant promise for managing obesity and associated metabolic disorders. This review synthesizes evidence on its modulation of AMP-activated protein kinase (AMPK) signaling, gut microbiota composition, lipid metabolism, and adipokine networks, elucidating how these actions converge to suppress adipogenesis and improve insulin sensitivity. Metabolomic profiling reveals critical shifts in bile acid metabolism, short-chain fatty acid production, and mitochondrial function. Recent studies also highlight berberine’s anti-inflammatory effects and regulatory influence on glucose homeostasis. Despite its promise, challenges in oral bioavailability and drug interactions necessitate the development of advanced delivery strategies. We further discuss nanoformulations and multi-omics approaches, which integrate data from genomics, transcriptomics, proteomics, and metabolomics, provide new insights into berberine’s mechanisms, and may guide personalized therapeutic applications. While promising, further studies are needed to validate these findings in humans and translate them into effective clinical strategies.

## 1. Introduction

Obesity is a complex, ongoing, and rapidly growing global health crisis of unprecedented scale. Marked by excessive fat buildup that poses a major health risk, obesity has evolved from a problem in wealthy nations to a widespread issue affecting countries across all income levels. Its roots run deep into the intricate relationships between biology, behavior, environment, and societal structures, making it one of the most significant public health challenges of the 21st century [1,2]. The World Health Organization reports that since 1990, the worldwide rate of obesity has more than doubled, while obesity among adolescents has increased fourfold [3]. Currently, over 1 billion people worldwide (about one in eight) are struggling with obesity, including around 650 million adults, 340 million teens, and 39 million kids [3]. If current trends hold, nearly one in five adults globally (18% of women and 21% of men) will be living with obesity by 2025 [4]. According to the National Health and Nutrition Examination Survey (NHANES), obesity rates among US adults rose to 41.9% in March 2020. Severe obesity affected 9.2% of adults during this time [5]. In Europe, the WHO’s 2022 regional report found that 59% of adults and nearly one-third of children were overweight or obese. The prevalence of adult obesity varies greatly, with rates above 30% in countries such as Türkiye, Malta, and Israel, as reported by the WHO Regional Office for Europe [6].

Obesity is a leading risk factor for a wide range of non-communicable diseases (NCDs), which greatly increases morbidity and mortality, lowers quality of life, and acts as a critical gateway to metabolic dysfunction. It is a primary driver of cardiovascular disease (CVD), including hypertension, dyslipidemia, coronary heart disease, heart failure, and stroke, as excess adipose tissue, particularly visceral fat, promotes inflammation, endothelial dysfunction, and insulin resistance [7]. The link between obesity and type 2 diabetes (T2DM) is exceptionally strong, since adipose tissue dysfunction impairs insulin signaling, leading to insulin resistance, with the majority of T2DM cases attributable to overweight and obesity [8,9]. Furthermore, obesity is associated with an increased risk of at least 13 different types of cancer, such as colon, rectum, gastric cardia, liver, gallbladder, pancreas, kidney, and esophageal adenocarcinoma, where mechanisms involve chronic inflammation, altered hormone metabolism, and elevated insulin levels [10]. Liver disease, specifically metabolic dysfunction-associated steatotic liver disease (MASLD) and its inflammatory form (MASH), is intimately linked to obesity and insulin resistance, potentially progressing to cirrhosis and liver cancer [11]. Finally, obesity is strongly and bidirectionally associated with mental health issues such as depression, anxiety, low self-esteem, and social stigmatization [12,13].

The complexity of obesity has spurred diverse intervention approaches. Modern anti-obesity medications (AOMs) target specific biological pathways implicated in appetite regulation and energy balance, with various mechanisms and indications. Glucagon-like peptide-1 (GLP-1) receptor agonists (e.g., liraglutide, semaglutide) mimic gut hormones to treat patients with obesity or who are overweight. Furthermore, glucose-dependent insulinotropic polypeptide (GIP)/glucagon-like peptide-1 (GLP-1) agonists (e.g., tirzepatide) offer a potent therapeutic option for adults meeting similar criteria. Other options include phentermine and topiramate, naltrexone and bupropion, oral hydrogel (which expands in the stomach and creates a feeling of fullness), and orlistat, which inhibits fat absorption in the intestine [14]. Despite their efficacy, challenges such as long-term safety, cost, access, and the universal need for sustained treatment in conjunction with diet and exercise persist. Concurrently, there is enduring interest in folk medicine and natural products for weight management.

However, berberine’s clinical use is limited by poor solubility and low oral bioavailability. To address these challenges, various carrier agents have been explored to enhance its absorption and therapeutic efficacy. Natural carrier systems such as liposomes, chitosan nanoparticles, and phytosomes have shown promise in improving berberine’s stability and bioavailability due to their biocompatibility and safety profiles [15].

**Figure 1:**
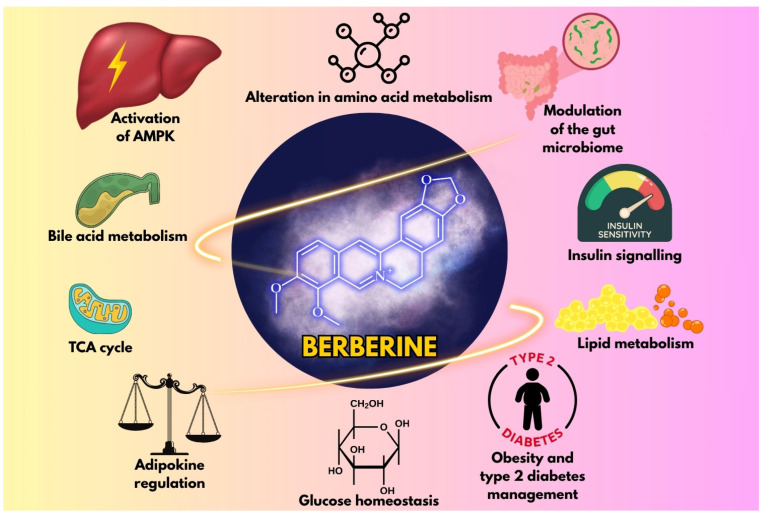
Illustration of the key mechanisms by which berberine influences metabolic health.

Berberine, an isoquinoline alkaloid found in plants such as *Berberis vulgaris* L. (barberry) and *Coptis chinensis* Franch. (goldthread), is a notable example that has been traditionally used in Ayurvedic and Chinese medicine [16,17]. Both preclinical and clinical studies suggest that berberine may modestly reduce body weight and improve metabolic parameters. This is achieved through the activation of AMP-activated protein kinase (AMPK), the modulation of the gut microbiome, and the regulation of lipid metabolism [18] (Figure 1).

This review paper aims to critically evaluate and synthesize the current scientific evidence supporting the anti-obesity potential of berberine, with a specific emphasis on understanding its distinct chemical structure, diverse natural origins, and the multifaceted molecular mechanisms underlying its metabolic effects.

## 2. Chemical Structure and Physicochemical Properties

**Figure 2:**
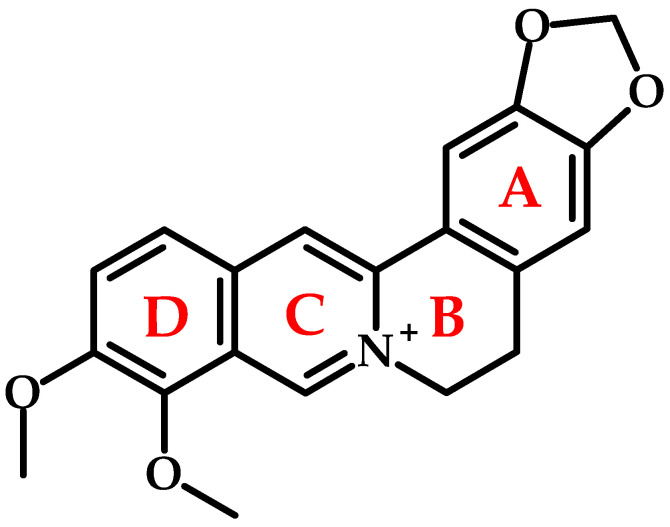
Chemical structure of berberine.

Berberine (Figure 2) is a naturally occurring, yellow-colored, cationic quaternary ammonium alkaloid. Its yellow color has been used in dyeing various materials, and berberine has a Color Index (CI) of 75,160. Chemically, it is defined as 5,6-dihydro-9,10-dimethoxybenzo[*g*]-1,3-benzodioxolo [5,6-*a*]quinolizinium, with the formula C_20_H_18_NO_4_^+^ and a molecular weight of 336.36 g/mol, although its chloride salt form (C_20_H_17_NO_4_HCl·2H_2_O, MW: 407.646 g/mol) is more commonly utilized.

Its distinct biological activity and fluorescence stem from a unique, planar fused tetracyclic ring system (A–D-rings; Figure 2) forming a benzodioxoloquinolizine core. Key structural features include a methylenedioxy group on the A-ring (C2-C3), a quaternary ammonium moiety on the C-ring, and methoxy groups at C9 and C10 on the D-ring. Interestingly, berberine exhibits fluorescence, with a maximum absorption wavelength of 350 nm and an emission wavelength of 530 nm in sodium dodecyl sulfate solution [19]. Also known as Natural Yellow 18, it is used to stain heparin in mast cells due to its fluorescent properties [20].

Berberine chloride demonstrates excellent solution stability, showing less than 5% degradation over six months across a wide pH range (1.2 to 9.0) at temperatures up to 40 °C [21], which is critical for oral formulation shelf-life and gastrointestinal transit integrity. Its crystalline state standard molar enthalpy of formation is −1335.2 kJ·mol^−1^, and its standard molar energy of combustion is −9536.2 kJ·mol^−1^ [19]. The melting point ranges from 145.1 to 146.7 °C.

Berberine chloride displays temperature-dependent solubility. It is readily soluble in hot water but sparingly soluble in cold water or ethanol, and essentially insoluble in nonpolar solvents (e.g., benzene, ether, chloroform) [22]. Solubility is also highly pH-dependent, and this pH-dependence directly impacts bioavailability, peaking at approximately 9.69 mM in phosphate buffer (pH 7.0, 37 °C) and decreasing significantly in acidic buffers. Solubility can be enhanced up to 4.5-fold via complexation with 2-hydroxypropyl-β-cyclodextrin (HPβCD), forming a 1:1 inclusion complex. Such solubility enhancement strategies are crucial for overcoming poor dissolution-limited absorption, a key barrier to therapeutic efficacy. While nonionic surfactants have negligible effects, ionic surfactants like sodium lauryl sulfate can reduce solubility, influencing excipient selection in formulations [21]. Table 1 summarizes critical physicochemical properties of berberine chloride.

## 3. Natural Occurrence and Sources

Berberine is a naturally occurring secondary metabolite found in many plant species from various plant families. As a bright yellow alkaloid, it is one of the most widely spread protoberberine compounds in the plant kingdom, with records of its occurrence on multiple continents and in different climates [23,24,25,26,27,28,29,30,31,32,33,34,35,36,37,38,39,40,41,42,43,44,45]. The detailed Table 2 below summarizes research findings from 2024 to 2025 on the distribution of berberine in various plant taxa.

This isoquinoline alkaloid shows a remarkable range of taxonomic diversity, occurring in at least six major plant families. The main sources include members of the Berberidaceae, Ranunculaceae, Menispermaceae, Malvaceae, Rutaceae, Annonaceae, and Vitaceae families. This broad phylogenetic distribution suggests that berberine biosynthesis evolved multiple times independently, with recent molecular studies confirming that distantly related lineages followed similar evolutionary paths.

The genus *Berberis* (Berberidaceae) exhibits substantial interspecific and intraspecific variation in berberine concentration, heavily dependent on the specific plant organ analyzed. *Berberis darwinii* Hook. roots yielded the highest recorded levels within this genus, reaching up to 26,482.20 µg/g dry weight, significantly surpassing concentrations found in stems (up to 6639.58 µg/g), seeds (up to 1181.75 µg/g), and leaves (up to 511.02 µg/g) [26]. Similarly, *B. thunbergii* DC. twigs contained notably higher berberine (0.364–0.676%) compared to *B. koreana* Palib. (0.042–0.067%) and *B. × ottawensis* “Superba” (0.103%) when extracted under identical ultrasonic conditions with 70% ethanol [38]. *B. vulgaris* root extracts prepared through hydro-ethanolic reflux yielded 111.06 mg/g using HPTLC quantification [35], whereas another paper showed a lower concentration of 78.95 µg/g dry extract [41]. Roots and stems of *B. vulgaris* extracted by Accelerated Solvent Extraction (ASE) with methanol yielded detectable berberine quantified by HPLC-ESI-Q-TOF-MS/MS, although specific concentrations were not reported [42].

Within the Ranunculaceae family, *Coptis* species, particularly their rhizomes and roots, emerge as vibrant sources. *C. chinensis* rhizomes exhibited berberine levels ranging from 15.2 to 46.2 mg/g dry weight using HPLC after ultrasound-assisted extraction (UAE) with various organic acids [27], up to 77.12 mg/g dry weight using a complex UHPLC-PDA method following DES-UA-MSPD extraction with betaine–acrylic acid [40]. Roots of the same species were reported to contain up to 10% berberine using DES-based coupled with UAE [39]. *C. teeta* Wall. rhizomes showed values of 212.18 ppm under optimized microwave-assisted extraction (MAE) conditions and 162.96 ppm with ultrasonic-assisted extraction (UAE) [32]. *Thalictrum foliolosum* DC. roots yielded 13.14 mg/g dry weight after UAE with a natural deep eutectic solvent (NADES) extraction [34], while a broader study on 57 batches of *Thalictrum* spp. stems and roots reported a wide range of 0.01 to 12.44 mg/g using LC-MS/MS after methanolic sonication [45].

*Phellodendron amurense* Rupr. (Rutaceae) bark was a source of berberine at the concentration of 0.243 mg/g of extract after microwave-assisted extraction [23], and a much higher 50.88 mg/g via UAE with aqueous malic acid [36]. *Tinospora cordifolia* (Willd.) Miers and *Coscinium fenestratum* (Gaertn.) Colebr. (Menispermaceae) stems were also confirmed sources. *T. cordifolia* stem concentrations included a reported 2.49% (*w*/*w*) via HPTLC after MAE [30], while other studies using HPLC-DAD or LC-MS/MS on hydro-alcoholic or UAE extracts did not report specific berberine values [24,25]. *C. fenestratum* stems and roots yielded 38.23 mg/g after extraction with 60% lactic acid [43]. *Annickia affinis* (Exell) Versteegh & Sosef (Annonaceae) stem bark contained approximately 0.02% *w*/*w* berberine quantified by LC-ESI-MS/MS after direct methanol extraction [44]. Moreover, some concentrations were noted for *Helicteres isora* L. fruits (21.37% of total area by GC-MS) [33], *Cayratia albifolia* C.L.Li roots [37], and *Mahonia bealei* (Fort.) Carr. leaves [28].

It underscores that berberine content is profoundly influenced by plant species, family (notably Berberidaceae and Ranunculaceae), and specific plant part (roots/rhizomes generally being richest). *Coptis* rhizomes and *Berberis* roots consistently rank among the highest natural sources. Modern extraction techniques are crucial for the efficient isolation of compounds. Ultrasound-assisted extraction (UAE) uses sound waves, microwave-assisted extraction (MAE) uses microwave energy, and accelerated solvent extraction (ASE) uses high pressure/temperature to enhance solvent penetration. Publications from the past two years reveal a strong trend toward using these techniques, often coupled with acidified solvents or novel solvents (DES/NADES), and advanced analytical methods like UHPLC-MS/MS, providing robust tools for efficient isolation and precise quantification. UAE was prevalent in the cited studies, employing solvents ranging from hydro–ethanolic mixtures [38,41] and organic acids [27,36] to innovative NADES/DES solvents [39,40]. Conventional methods of extraction are still used successfully. Such methods include reflux [29,41], boiling [37], and solvent shaking [44]. Interestingly, solvent composition, ratio, temperature, power, and time were critical optimized parameters that were universally optimized.

## 4. Pharmacological Mechanisms in Obesity and Diabetes

### 4.1. Impact on Obesity

**Figure 3:**
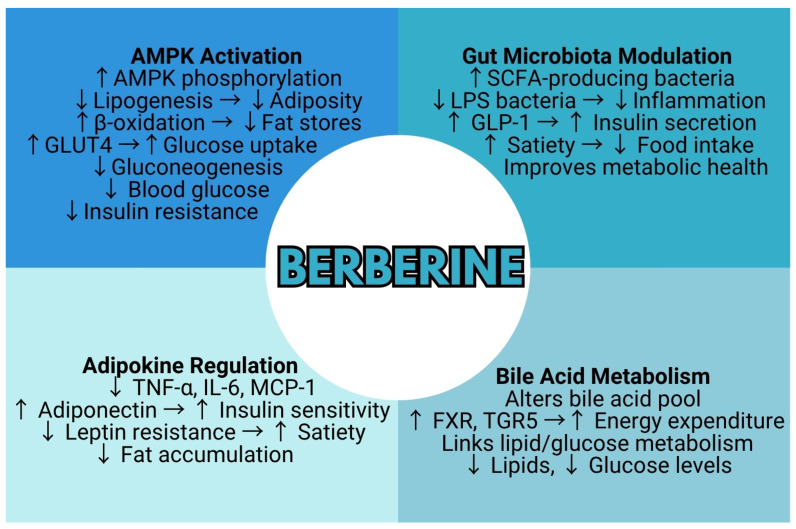
Integrative schematic of berberine’s molecular mechanisms in obesity and diabetes. Berberine activates AMP-activated protein kinase (AMPK), modulates gut microbiota, regulates adipokines, and alters bile acid metabolism, collectively reducing adiposity, improving insulin sensitivity, and decreasing lipid accumulation. Arrows indicate the direction of regulation or effect, with ↑ representing an increase or activation and ↓ representing a decrease or inhibition.

Berberine exhibits a broad spectrum of pharmacological activities that collectively influence key metabolic disturbances associated with obesity, insulin resistance, glucose homeostasis, and lipid metabolism, underscoring its potential as a multifaceted therapeutic agent in metabolic disorders. Its multidimensional actions are further discussed in this section, highlighting the diverse mechanisms through which berberine may exert its beneficial effects (Figure 3).

#### 4.1.1. Lipid Metabolism

When food energy consistently exceeds what the body can burn, two linked processes drive fat gain. First, dormant precursor cells are turned into new fat cells; second, those cells fill up with triglycerides. At the genetic level, the “foremen” that set this factory in motion are the transcription factors PPAR-γ and C/EBP-α. Berberine (BBR) short-circuits the very beginning of this programme: in classic 3T3-L1 cell assays, it cuts PPAR-γ and C/EBP-α expression almost in half, so far fewer precursors mature into adipocytes [46]. The same experiment shows a matching 50% drop in intracellular fat, illustrating how gene control translates directly into reduced lipid storage. Berberine not only prevents new fat cells from forming but also switches existing ones from storing energy to burning it. The key player here is AMP-activated protein kinase (AMPK)—a fuel gauge that senses when cellular ATP is low. Berberine nudges the ATP/AMP balance enough to activate AMPK; activated AMPK then inactivates acetyl-CoA carboxylase (ACC), lowering malonyl-CoA and opening the gate for fatty acids to pour into mitochondria for oxidation [47]. Independent groups have confirmed that the same AMPK-ACC cascade lowers lipogenesis in hepatocytes [48] and normalises liver fat in obese mice through both central and peripheral AMPK signaling [49]. The compound’s lipid-lowering reach goes beyond adipose tissue. In liver cells, BBR increases LDL-receptor mRNA and protein, thereby clearing LDL-cholesterol from the bloodstream by a mechanism distinct from statins [50]. Clinical trials echo these pre-clinical findings: treating patients with type 2 diabetes and mixed dyslipidemia with 1 g per day BBR for three months cut total cholesterol by about 18% and LDL-C by 21% [51]. A larger meta-analysis of eleven randomised controlled trials—874 participants in total—confirmed significant average reductions in total cholesterol (−0.61 mmol L^−1^), triglycerides (−0.50 mmol L^−1^), and LDL-C (−0.65 mmol L^−1^) with only mild gastrointestinal side effects [52]. Untargeted metabolomic profiling provides a panoramic snapshot of these molecular events in living animals. In high-cholesterol hamsters, berberine lowered circulating acyl-carnitines (signals of faster fatty-acid oxidation) and cholesteryl esters, while modestly raising specific bile acids—evidence that cholesterol was being routed into bile and excreted [53]. Complementary studies show that berberine’s persistent AMPK activation is tied to a sustained rise in the AMP/ATP ratio and an uptick in glycolysis, matching the overall energy-depleting signal required to keep fat burning switched on [54]. Berberine stops the “construction” of new fat depots and simultaneously turns existing depots into small furnaces. This dual action—genetic (fewer fat cells) and energetic (more fat burned)—explains why both laboratory models and human trials have consistently recorded lower body fat and blood lipid levels after berberine supplementation.

#### 4.1.2. Gut Microbiota

The adult intestine is home to an estimated 10^13^–10^14^ microorganisms drawn from thousands of bacterial species [55]. When these microbes ferment otherwise indigestible dietary fibre, they generate the short-chain fatty acids (SCFAs) acetate, propionate, and butyrate [56]. SCFAs are more than metabolic waste: butyrate is the preferred fuel for colonocytes and supports mucosal energy needs, whereas all three acids tighten epithelial junctions and stimulate mucin secretion, thereby reinforcing the intestinal barrier [57,58]. Beyond the gut, SCFAs activate free-fatty-acid receptors on enteroendocrine cells, provoking the release of PYY and GLP-1, hormones that quell appetite and enhance insulin sensitivity [59]. A sustained high-fat diet (HFD) perturbs this balance. It selectively depletes many SCFA-producing taxa and enriches Gram-negative Proteobacteria; the resulting surge in lipopolysaccharide (LPS) compromises tight-junction integrity, leaks into the bloodstream, and kindles low-grade “metabolic endotoxemia” [60,61]. The loss of SCFA supply, coupled with LPS-driven inflammation, undermines barrier function, blunts satiety signaling, and feeds into weight gain and insulin resistance. Early animal work put berberine on the microbiome map. In rats fattened on a high-fat diet, berberine shifted the bacterial community toward SCFA-producing genera such as *Allobaculum*, *Blautia,* and *Butyricicoccus*, cut overall microbial diversity, and—importantly—lowered the animals’ adiposity index (a measure of total body fat) [62]. A companion redundancy-analysis linked 134 berberine-responsive bacterial “operational taxonomic units” (OTUs) to changes in body weight, leptin, and insulin, drawing a straight line from microbiome rewiring to host metabolism. An earlier study from the same group had already shown that these compositional shifts coincide with higher caecal SCFA levels and a partial reversal of LPS-driven inflammation [63].

Since 2020, multi-omics approaches have deepened this picture. In a mouse model of metabolic syndrome, berberine not only enriched SCFA producers but also restored the tight-junction proteins ZO-1 and occludin, reduced serum LPS, and quieted hepatic inflammatory genes—changes that paralleled improvements in weight and lipid profiles [64]. Attention has turned to species-level “keystone” taxa. Clinical work in hyperlipidemic patients showed that the abundance of *Blautia* and *Alistipes* at baseline could predict who would respond best to berberine’s cholesterol-lowering action; antibiotic depletion or fecal-transplant studies confirmed that the gut microbiota is necessary and sufficient for this effect [65]. Follow-up mechanistic experiments identified Blautia producta as a berberine-enriched commensal capable of upregulating hepatic LDL receptors, lowering serum LDL-C, and, through its butyrate output, contributing to SCFA pools [66]. Berberine also influences how microbes transform bile acids, detergent-like molecules that serve as metabolic hormones. By selectively promoting bile-salt-hydrolase-inhibiting species, berberine shifts the bile-acid pool toward forms that activate the intestinal receptor FXR and the systemic receptor TGR5, both linked to better glucose tolerance and energy expenditure [64].

Importantly, gut-centered actions mesh with classic pharmacology: combining berberine with metformin in diabetic mice produced a greater fall in blood glucose than either drug alone and generated a distinct microbiota signature—higher *Akkermansia* and *Verrucomicrobia*, and lower Proteobacteria—alongside higher circulating metformin levels [67]. These findings suggest that manipulating the microbiome may help fine-tune berberine dosing or uncover synergistic drug pairs in metabolic disease.

In summary, berberine remodels the gut ecosystem in three concerted steps: 1. It enriches SCFA-producing bacteria, boosting metabolites that curb appetite and inflammation. 2. It suppresses LPS-laden Gram-negative taxa, tightening the intestinal barrier and lowering systemic inflammatory tone. 3. It steers bile-acid conversions toward hormonal profiles that favor leanness and insulin sensitivity. Together, these microbial shifts create a biochemical environment that echoes and amplifies berberine’s direct effects on lipid and glucose metabolism, reinforcing its promise as a diet-compatible tool for obesity management.

#### 4.1.3. Adipokine Regulation

Adipose tissue, traditionally viewed as a passive fat storage site, is now recognized as a highly active endocrine organ, secreting a variety of bioactive peptides known as adipokines. Among these, leptin and adiponectin are two of the most studied due to their significant roles in energy homeostasis, metabolic regulation, and inflammation. Alongside these, inflammatory cytokines such as tumor necrosis factor-alpha (TNF-α), interleukin-6 (IL-6), and C-reactive protein (CRP) are significantly influenced by changes in adipose tissue dynamics and play a central role in the development of obesity-related complications. Leptin is primarily involved in appetite regulation and energy balance. Its secretion increases with fat mass, acting on the hypothalamus to suppress food intake and increase energy expenditure. However, in obesity, leptin resistance often develops, characterized by elevated circulating levels of leptin without corresponding satiety signals. This resistance blunts its physiological role, contributing to continued weight gain and metabolic dysregulation [68]. Adiponectin, in contrast, exhibits inverse behavior with respect to adiposity. It enhances insulin sensitivity and exhibits anti-inflammatory and anti-atherogenic properties. Lower adiponectin levels are frequently observed in individuals with obesity, type 2 diabetes, and cardiovascular disease, suggesting its protective role against metabolic dysfunction [69].

The secretion of inflammatory cytokines from adipose tissue, particularly from infiltrating immune cells like macrophages, is another hallmark of dysfunctional adipose tissue. Increased levels of TNF-α and IL-6 in obesity contribute to systemic inflammation, insulin resistance, and the progression of metabolic syndrome. These cytokines not only interfere with insulin signaling pathways but also suppress adiponectin production, creating a feedback loop that exacerbates metabolic disturbances [70].

The balance between these adipokines and cytokines is tightly regulated and highly sensitive to lifestyle factors, including diet, physical activity, and pharmacological interventions. Weight loss, for example, has been shown to decrease leptin and inflammatory cytokine levels while increasing adiponectin concentrations, thereby improving metabolic outcomes. The regulation of leptin, adiponectin, and inflammatory cytokines underscores the complex interplay between adipose tissue and systemic metabolic health. Targeting these adipokines offers promising therapeutic potential in managing obesity and its associated disorders.

Adipose tissue is not a passive energy store; it secretes a spectrum of protein messengers—adipokines—that influence appetite, inflammation, and insulin responsiveness throughout the body. During weight gain, this secretory profile drifts in an unfavorable direction. Circulating leptin, which usually signals satiety to the brain, rises in parallel with fat mass; yet, central leptin receptors become desensitized, a state termed leptin resistance [63]. At the same time, adiponectin, an anti-inflammatory adipokine that promotes glucose uptake and fatty-acid oxidation, falls sharply. The imbalance is compounded by chemokines, such as monocyte-chemoattractant protein-1 (MCP-1), which draw immune cells into adipose depots, fueling chronic, low-grade inflammation [53].

In rodent models of diet-induced obesity, oral berberine consistently reverses this endocrine drift. Eight to twelve weeks of treatment at 100–200 mg kg^−1^ day^−1^ lowers plasma leptin and MCP-1 while restoring adiponectin when normalized to fat mass [63]. Untargeted metabolomics links these hormonal shifts to a rise in glutathione and a decline in arachidonic-acid metabolites—biochemical signatures of reduced oxidative stress and dampened eicosanoid-driven inflammation [53]. More recent multi-omics studies confirm that berberine enhances antioxidant defenses and suppresses lipid peroxidation in parallel with normalizing adipokines [64].

Formulation improvements appear to magnify these effects. A co-crystal combining berberine with ibuprofen, which increases intestinal absorption three-fold, boosted serum adiponectin by 60% and lowered MCP-1 expression in adipose tissue more potently than native berberine in obese db/db mice [71].

Several molecular pathways converge to explain the endocrine reset. Berberine activates the AMPK–SIRT1 axis, which suppresses the transcription factor NF-κB and thereby limits MCP-1 production [72]. It also alleviates endoplasmic reticulum stress in adipocytes, a condition known to blunt leptin signaling, and curtails 12/15-lipoxygenase activity, reducing pro-inflammatory lipid mediators [53].

The same direction is being pointed to by early clinical data, even if the scale is limited. In adults with metabolic syndrome, twelve weeks of berberine (500 mg three times daily) reduced the leptin-to-adiponectin ratio by nearly one-third, and this change was associated with improved insulin sensitivity [73]. A randomised trial in patients experiencing antipsychotic-associated weight gain found that adjunctive berberine (600 mg day^−1^) halved serum leptin, suggesting improved leptin responsiveness, although adiponectin was unchanged [74]. In poorly controlled type 2 diabetes, adding berberine (1 g day^−1^) to metformin lowered MCP-1 and IL-6 and restored adiponectin to near-normal levels while improving glycemic control [67].

Collectively, the pre-clinical and emerging clinical evidence indicates that berberine recalibrates adipose-derived signals on three fronts: it reduces pro-inflammatory cues (lower leptin and MCP-1), restores anti-inflammatory tone (higher adiponectin), and improves the oxidative milieu that underpins adipokine secretion. Through these integrated actions, modest weight reductions achieved under berberine are translated into disproportionately large gains in insulin sensitivity and systemic metabolic health.

### 4.2. Role in Diabetes and Insulin Resistance

#### 4.2.1. Glucose Homeostasis

Maintaining stable blood-glucose levels hinges on two tightly linked processes. First, glucose must be cleared from the circulation into peripheral tissues, chiefly skeletal muscle and adipose tissue, via insulin-stimulated translocation of the GLUT4 transporter to the cell surface. In obesity and type 2 diabetes, this trafficking step falters; impaired GLUT4 delivery is now recognized as a core molecular lesion of insulin resistance [75]. Second, the liver must restrain its own production of glucose through gluconeogenesis during the fasted state. In diabetes, that restraint is lost: hepatic expression of gluconeogenic enzymes such as phosphoenolpyruvate carboxykinase and glucose-6-phosphatase remains inappropriately high, so the liver continues to add glucose to the bloodstream even when levels are already elevated, driving fasting hyperglycemia [76].

In summary, maintaining stable blood glucose levels relies on two processes working together: the efficient uptake of glucose into the tissues of the body (mostly muscle and fat) and the controlled release of glucose from the liver through a process called gluconeogenesis. In obesity and type 2 diabetes, both arms fail—the insulin signal that moves the GLUT4 transporter to the cell surface is blunted, and the liver continues to manufacture glucose even when circulating levels are already high.

Recent work places berberine at the crossroads of these abnormalities. In skeletal muscle and cultured myotubes, the alkaloid raises the AMP–ATP ratio and thereby activates the cellular energy sensor AMP-activated protein kinase (AMPK). Activated AMPK, in turn, drives GLUT4 molecules to the plasma membrane, boosting glucose uptake independently of the classic PI3K–Akt insulin cascade [77]. Because this route bypasses the insulin receptor, it remains functional in insulin-resistant states—a key advantage over therapies that rely solely on enhancing insulin signaling.

Berberine’s influence extends to the liver, where it dampens gluconeogenesis. Both in hepatocyte culture and in diabetic rodent models, the compound reduces transcription of the rate-limiting enzymes phosphoenolpyruvate carboxykinase (PEPCK) and glucose-6-phosphatase (G6Pase), leading to appreciably lower fasting glucose concentrations. Two complementary signaling routes have been described. First, activation of the upstream kinase LKB1 funnels through AMPK and TORC2 to silence gluconeogenic genes [78]. Second, berberine modulates an AKT/MAPK/NO/cGMP/PKG axis, again converging on PEPCK-G6Pase repression and reinforcing the hypoglycemic effect [79].

These molecular findings translate into measurable clinical benefits. In a 12-week, randomized trial of the gut-targeted berberine-ursodeoxycholate co-crystal (HTD1801) involving 113 adults with type 2 diabetes, the higher dose (1 g twice daily) lowered HbA1c by 0.5 percentage points relative to placebo and produced parallel reductions in fasting glucose; exploratory biomarker work confirmed AMPK activation and suppressed gluconeogenic gene signatures [80].

In summary, berberine tackles glucose dysregulation on two fronts: 1. peripheral uptake—it activates AMPK in muscle and adipose tissue, moving GLUT4 to the cell surface and allowing glucose to enter cells even when insulin signaling is impaired; and 2. hepatic output—it switches off the key enzymes of gluconeogenesis via AMPK-centered (and AKT/MAPK-linked) pathways, trimming the liver’s contribution to blood glucose. By synchronising these effects, berberine restores a more balanced glucose economy, an action pattern that underpins its growing appeal as an adjunct—or, in some formulations, an alternative—to current antidiabetic medications.

#### 4.2.2. Insulin Signaling

Efficient insulin action rests on two molecular events: (1) insulin must recruit the glucose-transporter GLUT4 to the surface of skeletal-muscle and adipose cells so that glucose leaves the bloodstream, and (2) hepatic production of glucose through gluconeogenesis must be dialled down when systemic concentrations are already high. In obesity and type 2 diabetes, these safeguards unravel the following: free fatty acids and inflammatory cues stimulate serine/threonine kinases that phosphorylate insulin-receptor substrate-1 (IRS-1) on inhibitory serine sites, disconnecting it from phosphatidyl-inositol-3-kinase (PI3K) and blocking downstream Akt activation; at the same time, gluconeogenic enzymes in the liver remain inappropriately active [75].

BBR restores this broken circuitry at several checkpoints. In hepatocytes challenged with TNF-α, BBR inhibited the upstream MEKK1/MEK–ERK1/2 module, preventing ERK-driven serine phosphorylation of IRS-1 and, thereby, re-establishing IRS-1 tyrosine phosphorylation, PI3K coupling, and Akt activity; glucose uptake normalized in parallel [81]. In skeletal muscle, high-fructose feeding normally blunts Akt and GLUT4 translocation, yet eight weeks of oral BBR reversed these defects while raising the AMP/ATP ratio and activating AMPK—again without relying on canonical PI3K-Akt insulin signaling [77].

Inflammation-linked kinases provide a second entry point. In high-fat-diet mice, BBR lowered hepatic and adipose phosphorylation of IKK-β (Ser^181^) and curtailed NF-κB nuclear translocation, changes that coincided with reduced IRS-1^Ser phosphorylation and improved insulin-stimulated Akt activity [64]. Complementary work in sarcopenic mice shows that BBR also activates a SIRT1-mediated mitophagy axis, relieving oxidative stress, suppressing IKKβ/NF-κB tone, and reinstating insulin responsiveness in ageing muscle [82].

Across tissues, these node-specific actions translate into a coherent network response. A systems-biology review that collated multi-omics and docking data for >40 natural alkaloids places BBR among the most consistent enhancers of the InsR–IRS–PI3K–Akt axis in liver, muscle, and adipose tissue, with reported gains in glucose uptake ranging from 20% in vitro to 45% in vivo [83]. An even broader 2024 database-driven meta-analysis of >300 BBR targets confirms frequent high-affinity docking to kinases that gate insulin signaling—IKKβ, JNK, and mTORC2—and highlights the compound’s dual ability to quench inflammatory serine kinases while sparing, or even amplifying, canonical tyrosine-kinase cascades [84].

Taken together, contemporary evidence indicates that berberine rescues insulin signaling at its most vulnerable links—IRS-1 integrity, PI3K engagement, and Akt-driven GLUT4 trafficking—while dampening the inflammatory and stress-kinase landscape that erodes these links in the first place. This multi-node repair explains why berberine can lower fasting glucose and improve whole-body insulin sensitivity, even when insulin receptor activity per se is not increased, making the alkaloid a mechanistically distinct complement to existing antidiabetic agents.

#### 4.2.3. Mitochondrial Function

Mitochondria serve as the cell’s principal sites of ATP generation, transforming nutrients into usable energy while modulating intracellular reactive oxygen species (ROS) levels [85]. Both the number of mitochondria and their functional integrity decline in obesity and type 2 diabetes, a change strongly implicated in the development of insulin resistance and ectopic lipid accumulation [86]. A growing body of work shows that berberine counteracts this mitochondrial deficit on two fronts: it stimulates mitochondrial biogenesis, the synthesis of fresh organelles, and it promotes mitophagy, the selective clearance of damaged ones—effects that together restore oxidative capacity and redox balance [82,87,88].

A unifying upstream event is the activation of the AMP-activated protein kinase (AMPK)–SIRT1–PGC-1α axis. In diet-induced sarcopenic mice, eight weeks of oral BBR restored grip strength, doubled mitochondrial DNA copy number, and raised maximal respiratory capacity; pharmacological blockade of SIRT1 abolished these benefits, pinpointing the SIRT1–PGC–1α module as indispensable [82]. Similar results emerge in isolated PINK1-knock-out fibroblasts, where BBR sparks AMPK-dependent PINK1/Parkin mitophagy while up-regulating the biogenic regulators NRF-1 and TFAM, normalizing ATP output without compromising membrane potential [88].

Tissue-specific studies corroborate this dual action. In the liver, BBR improves insulin sensitivity by enhancing SIRT1-directed expression of Opa1, thereby harmonizing mitochondrial fusion–fission dynamics and boosting oxidative phosphorylation efficiency [87]. Cardiac models of heart failure with preserved ejection fraction display depressed AMPK and swollen, ROS-leaking mitochondria; four weeks of BBR reinstated AMPK/PGC-1α signaling, corrected TFAM-driven biogenesis, and limited mitochondrial apoptosis, culminating in better diastolic function [89]. Low-dose BBR also shields cardiomyocytes from doxorubicin toxicity by dissociating the Bcl–xL–Beclin–1 complex, unleashing mitophagy and quenching ROS [90].

Structural analogues reinforce the story. Tetrahydroberberrubine, a more lipophilic derivative, delayed heart ageing in mice by stabilising PHB2 mRNA, a pivotal mitophagy receptor, and thereby accelerating turnover of senescent mitochondria [91]. Such findings highlight the therapeutic headroom available through formulation or chemical modification.

A systems biology survey of >300 BBR targets places mitochondrial regulators at the top of the affinity list and reports convergent activation of the AMPK–SIRT1–PGC–1α network in liver, muscle, heart, and adipose tissue [84]. Collectively, this evidence positions BBR as a dual-action restorer of mitochondrial quantity and quality. By seeding fresh mitochondria and removing defective ones, BBR elevates oxidative capacity and reduces oxidative stress, creating the metabolic environment needed for improved insulin signaling and lipid handl

## 5. Metabolomic Insights into Berberine’s Therapeutic Effects

### 5.1. Obesity and the Anti-Obesity Effects of Berberine

Obesity is characterized by excessive fat accumulation resulting from an imbalance between energy intake and expenditure. This condition is a significant risk factor for metabolic disorders, including type 2 diabetes, cardiovascular disease, and non-alcoholic fatty liver disease. Berberine has demonstrated significant anti-obesity effects, primarily through modulation of lipid metabolism and bile acid (BA) pathways [92,93].

#### 5.1.1. Lipid Profile Modulation

BBR has been shown to reduce serum triglycerides (TG) and low-density lipoprotein cholesterol (LDL-C) levels, thereby improving lipid profiles in obese models. Mechanistically, BBR activates peroxisome proliferator-activated receptor delta (PPAR-δ), which enhances mitochondrial fatty acid oxidation and reduces lipid accumulation in adipocytes. In high-fat diet (HFD)-induced obese mice, BBR administration significantly reduced body weight gain and adipose tissue mass. These effects were markedly diminished upon pharmacological inhibition of PPAR-δ, underscoring this receptor’s pivotal role in the lipid-lowering action of BBR [94].

Moreover, BBR enhances basal triglyceride lipolysis in adipocytes and suppresses adipogenesis by downregulating the expression of lipogenic transcription factors, including liver X receptor alpha (LXRα), peroxisome proliferator-activated receptor gamma (PPAR-γ), and sterol regulatory element-binding protein-1c (SREBP-1c). These effects collectively inhibit preadipocyte differentiation and lipid droplet accumulation, further supporting BBR’s anti-obesity potential [94].

In a clinical trial involving hyperlipidemic patients, oral administration of BBR (500 mg twice daily) for 3 months significantly reduced serum cholesterol and triglyceride levels, comparable to standard statin therapy, but with fewer reported side effects [50]. These findings provide translational relevance to the lipid-lowering effects observed in preclinical studies.

To sum up, berberine lowers serum levels of triglycerides (TG), total cholesterol (TC), and low-density lipoprotein cholesterol (LDL-C). It enhances LDL receptor (LDLR) expression in the liver, which promotes hepatic clearance of LDL-C [50]. BBR also downregulates lipogenic genes such as SREBP-1c and FASN, inhibiting lipid synthesis [92]. In addition, it activates AMP-activated protein kinase (AMPK) and peroxisome proliferator-activated receptor delta (PPAR-δ), leading to increased fatty acid oxidation and reduced fat accumulation [94].

#### 5.1.2. Bile Acid Metabolism

Bile acids are not only crucial for dietary lipid digestion but also serve as important signaling molecules involved in the regulation of glucose and energy homeostasis via nuclear and membrane receptors such as farnesoid X receptor (FXR) and Takeda G-protein-coupled receptor 5 (TGR5). BBR modulates BA metabolism primarily through alterations in gut microbiota composition, particularly enhancing the abundance of bacteria capable of converting primary to secondary BAs [95].

This remodeling of the BA pool composition subsequently activates FXR and TGR5 signaling, leading to improved insulin sensitivity, increased energy expenditure, and reduced hepatic steatosis. In mouse models, BBR increased the expression of the bile salt export pump (BSEP) and small heterodimer partner (SHP), downstream targets of FXR activation, which play key roles in maintaining BA homeostasis [96].

Additionally, BBR upregulates cholesterol 7α-hydroxylase (CYP7A1), the rate-limiting enzyme in BA synthesis from cholesterol, thereby promoting cholesterol clearance and reducing circulating LDL-C levels. This enzymatic shift not only aids in lipid reduction but also contributes to the anti-atherogenic properties of BBR [97].

### 5.2. Diabetes: Shifts in Amino Acids (e.g., Branched-Chain Amino Acids), TCA Cycle Intermediates, and Gut Microbiota-Derived Metabolites

Diabetes mellitus encompasses a group of metabolic diseases characterized by chronic hyperglycemia resulting from defects in insulin secretion, insulin action, or both. Type 1 diabetes mellitus (T1DM) is primarily caused by the autoimmune destruction of pancreatic β-cells, leading to absolute insulin deficiency [98]. In contrast, type 2 diabetes mellitus (T2DM) involves insulin resistance and a relative insulin deficiency [99]. Both types are associated with profound metabolic disturbances extending beyond glucose metabolism. Emerging evidence highlights significant alterations in amino acid profiles [100], disruptions in mitochondrial function as seen through tricarboxylic acid (TCA) cycle intermediates [101], and dysregulation of gut microbiota-derived metabolites [102] in both T1DM and T2DM. These changes offer new insights into disease pathophysiology and potential therapeutic targets.

#### 5.2.1. Alterations in Amino Acid Metabolism

Elevated levels of branched-chain amino acids (BCAAs)—leucine, isoleucine, and valine—are consistently observed in T2DM and are associated with insulin resistance and impaired glucose metabolism. Reduced catabolism of BCAAs has been documented, resulting in their accumulation and contributing to metabolic inflexibility [103]. This is primarily due to the downregulation of enzymes such as branched-chain α-keto acid dehydrogenase.

In addition, alterations in arginine metabolism are noted in diabetes. Increased activity of the enzyme arginase leads to higher levels of ornithine and proline, which may impair nitric oxide availability and promote endothelial dysfunction, contributing factors to vascular complications in diabetic patients [104].

#### 5.2.2. Disruptions in TCA Cycle Intermediates

The TCA cycle is essential for cellular respiration and energy production. In T2DM, several studies report reductions in key intermediates such as citrate, α-ketoglutarate, and succinate, particularly in skeletal muscle tissue [101]. This suggests mitochondrial dysfunction and a shift toward anaerobic glycolysis, which limits ATP production and promotes lactate accumulation.

Diabetic individuals also exhibit a reliance on fatty acid and amino acid oxidation for energy, rather than glucose oxidation. This metabolic shift exacerbates insulin resistance and contributes to lipid accumulation in non-adipose tissues [101].

#### 5.2.3. Gut Microbiota-Derived Metabolites and Their Impact

The gut microbiota plays a critical role in modulating host metabolism. In diabetes, gut dysbiosis alters the production of bioactive microbial metabolites, including short-chain fatty acids (SCFAs), bile acids, and trimethylamine N-oxide (TMAO). Reduced production of butyrate, a key SCFA, is associated with impaired gut barrier function and systemic inflammation, both of which contribute to insulin resistance [105]. In contrast, elevated circulating levels of TMAO in diabetes have been linked to increased cardiovascular risk, due to its pro-inflammatory and pro-atherogenic effects [105].

Both type 1 and type 2 diabetes are characterized by distinct and overlapping metabolic perturbations, including altered amino acid metabolism, disrupted tricarboxylic acid (TCA) cycle function, and imbalances in gut microbiota-derived metabolites. These shifts contribute to the progression of insulin resistance, mitochondrial dysfunction, and chronic inflammation, providing mechanistic insights that may inform future therapeutic strategies.

### 5.3. Multi-Omics Integration: Linkages Between Metabolomics, Transcriptomics, and Proteomics Data

Understanding the multifaceted therapeutic effects of BBR necessitates a systems-level approach that transcends the study of isolated molecular pathways. Integrating multi-omics datasets—particularly metabolomics, transcriptomics, and proteomics—has proven to be a powerful strategy for unraveling the complex biological networks and regulatory circuits modulated by BBR across different tissues and disease contexts.

#### 5.3.1. Holistic Insights Through Multi-Omics Platforms

Multi-omics approaches enable the simultaneous interrogation of metabolic flux, gene expression, and protein abundance, providing a comprehensive understanding of how BBR orchestrates systemic physiological changes. In metabolic diseases such as type 2 diabetes mellitus (T2DM) and obesity, BBR has been shown to coordinate responses at multiple molecular levels, restoring metabolic balance, reducing inflammation, and improving insulin sensitivity [106,107,108].

#### 5.3.2. Berberine in Inflammatory Bowel Disease: A Multi-Omics Paradigm

One of the clearest examples of multi-omics integration in BBR research comes from studies on inflammatory bowel disease (IBD). In a murine model of ulcerative colitis, Yu et al. [109] employed a combination of metagenomics, metabolomics, and transcriptomics to demonstrate that BBR modulated gut microbiota composition—enriching beneficial taxa like *Lactobacillus*—and restored the expression of tight junction proteins (e.g., ZO-1, occludin), thereby reinforcing intestinal barrier integrity. Concurrently, significant shifts in bile acid metabolism were observed, suggesting that BBR’s efficacy arises from the interplay between microbial, metabolic, and epithelial pathways [109].

Similarly, Yang et al. [110] employed a multi-omics approach, integrating 16S rDNA sequencing, serum metabolomics, and transcriptomics, to investigate the effects of BBR on the intestinal microbiome and serum metabolome in ulcerative colitis. Their findings revealed that BBR ameliorated the community of intestinal microbiota and significantly promoted the abundance of beneficial bacteria such as *Muribaculaceae* and *Akkermansia*. Furthermore, BBR modulated inflammation-related metabolites and metabolic pathways in serum, indicating its role in restoring metabolic homeostasis [110].

#### 5.3.3. Transcriptome–Proteome Interplay in Energy Metabolism

Transcriptomic and proteomic analyses have revealed that BBR modulates multiple pathways involved in mitochondrial function and energy metabolism. For instance, integrated omics profiling has shown that BBR upregulates peroxisome proliferator-activated receptor gamma coactivator 1-alpha (PGC-1α) and carnitine palmitoyltransferase 1A (CPT1A), key regulators of fatty acid β-oxidation, while downregulating lipogenic genes such as SREBP-1c and FASN. These changes align with increased mitochondrial biogenesis and oxidative phosphorylation, consistent with improved metabolic phenotypes in BBR-treated models. Additionally, proteomic data have confirmed alterations in enzymes of the tricarboxylic acid (TCA) cycle and glycolysis, supporting the hypothesis that BBR redirects cellular energy metabolism toward improved glucose utilization and decreased lipid storage [106,111].

#### 5.3.4. MAPK and Inflammatory Pathways: Multilayered Regulation

A study employing transcriptomics and phosphoproteomics revealed that BBR significantly suppresses the MAPK signaling cascade—including ERK, JNK, and p38 pathways—thereby attenuating the expression of pro-inflammatory cytokines in chronic atrophic gastritis models. This multilayered regulation at both the transcriptional and post-translational levels suggests that BBR not only dampens inflammation but also reprograms the cellular stress response machinery [112]. Studies indicate that BBR suppresses the activation of these kinases, leading to a decrease in the expression of pro-inflammatory cytokines, including TNF-α, IL-1β, and IL-6 [112]. This modulation occurs through both transcriptional and post-translational mechanisms, suggesting a multilayered regulatory effect on the inflammatory response.

For instance, in models of gastric mucosal inflammation, BBR inhibited the phosphorylation of ERK, JNK, and p38, which was associated with reduced cytokine expression and improved mucosal barrier integrity [112]. Furthermore, BBR has been shown to influence the expression of stress response-related genes, including HO-1 and Nrf2, indicating a role in enhancing cellular defense mechanisms [50].

**Figure 4:**
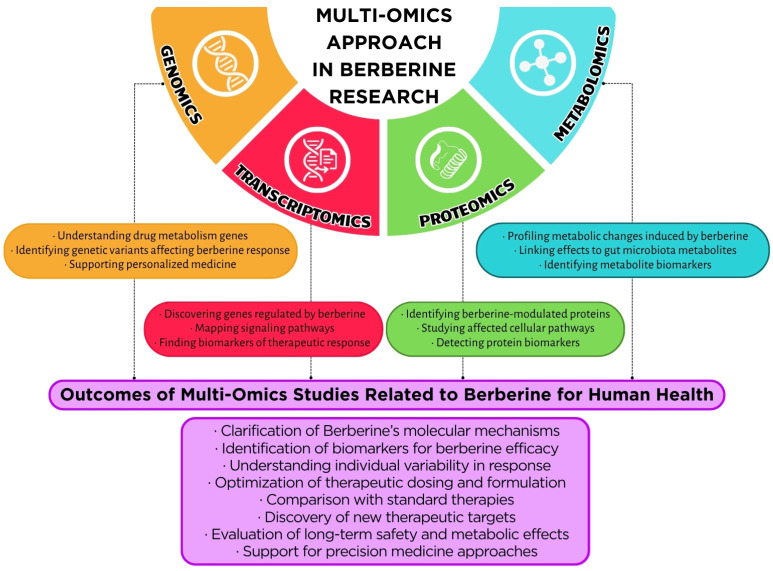
Schematic illustrating the multi-omics approach in berberine research. Genomics, transcriptomics, proteomics, and metabolomics data reveal the systemic effects of berberine, enabling the discovery of biomarkers and the development of personalized metabolic therapies.

Although direct studies using combined transcriptomic and phosphoproteomic approaches to evaluate BBR’s effects on the MAPK pathway remain limited, available data support its potential to influence this pathway via modulation of gene expression and kinase activity. Further research employing advanced omics technologies is warranted to comprehensively understand these interactions and confirm the molecular mechanisms underlying BBR’s effects (Figure 4).

## 6. Safety, Bioavailability, and Future Directions

### 6.1. Pharmacokinetics, Toxicity, and Side Effects

Berberine has a remarkably wide range of health benefits. These include regulating glucose and lipid metabolism, as mentioned earlier, as well as fighting infections, reducing inflammation, and showing potential for protecting brain and heart health [113]. Although berberine shows excellent promise as a therapeutic, several major obstacles hinder its widespread use in clinical settings. The main challenges are its unfavorable pharmacokinetic profile, which results in very low bioavailability, and the need for a thorough understanding of its long-term safety profile, particularly regarding interactions with other medications and its use in specific populations [114]. Overcoming these limitations through innovative research is crucial to harnessing the full potential of berberine and expanding its established uses.

One major limitation of berberine’s clinical use is its very low absorption rate in the body, which is usually estimated to be less than 1% when taken orally [115]. Moreover, berberine is poorly absorbed across the intestinal epithelium. Its chemical structure, featuring a quaternary ammonium group, contributes to low aqueous solubility and poor passive diffusion through the lipid membranes of the intestinal cells [15]. Only a small part of the berberine that is absorbed enters the portal vein, where it undergoes significant metabolism in the liver before entering systemic circulation. The primary reactions involved are Phase II conjugation reactions, specifically glucuronidation, which forms metabolites such as berberrubine glucuronide, and sulfation. These metabolites often have lower pharmacological activity than the original berberine compound [116]. Berberine and its metabolites are primarily eliminated from the body via renal excretion (urine) and biliary excretion (feces). Its elimination half-life is relatively short, typically ranging from a few to several hours [117]. This necessitates frequent dosing to maintain potentially therapeutic plasma concentrations.

The gut microbiota plays a crucial role in overcoming berberine’s low oral bioavailability (<1%). Cheng et al. [118] confirmed that despite poor solubility and absorption, berberine enters systemic circulation through bidirectional interactions with gut bacteria. Importantly, anaerobic bacteria (e.g., *Enterobacter cloacae*, *Escherichia coli*) convert berberine into dihydroberberine via bacterial nitroreductase activity [119], a reduced form that shows 5-fold higher intestinal absorption because of increased lipophilicity and decreased positive charge [119]. After absorption, dihydroberberine quickly re-oxidizes non-enzymatically back to active berberine in intestinal tissues [119], acting as a “stealth transporter” that bypasses P-glycoprotein efflux. This microbial biotransformation greatly boosts systemic berberine levels [119], which explains its strong pharmacological effects despite low plasma concentrations [119]. Meanwhile, berberine alters the gut ecosystem: it encourages beneficial SCFA-producing bacteria (e.g., *Lachnospiraceae*, *Blautia*) and suppresses harmful pathogens [118,120]. Interestingly, *Blautia* species further metabolize berberine into bioactive demethylated compounds (e.g., thalifendine) through acetogenesis [120], aiding therapeutic effects and promoting cross-feeding that increases butyrate production [118,120]. Antibiotic treatment that disrupts gut microbiota reduces both dihydroberberine formation and demethylation pathways, leading to lower systemic berberine levels and weaker glucose-lowering and lipid-regulating effects [118,119].

Berberine is generally regarded as safe for short to medium-term use in healthy adults at typical therapeutic doses (commonly 500–1500 mg per day, administered in divided doses) [121]. It shows relatively low acute toxicity. Research in mice found an LD_50_ of pure berberine to be 329 mg/kg when given orally and 23 mg/kg when given intraperitoneally [122]. The most commonly reported side effects are related to the digestive system, likely due to the high concentration of the substance in the gut and its antimicrobial properties. Symptoms like diarrhea, constipation, gas, stomach cramps, and nausea are common, especially at higher doses or when treatment first starts. These symptoms often decrease with continued use or a lower dose [123].

Berberine inhibits several CYP enzymes, primarily CYP2D6, CYP2C9, and CYP3A4, which can significantly increase blood levels and the risk of toxicity for drugs that rely on these pathways. This includes antidepressants, statins, and immunosuppressants. Berberine also blocks P-glycoprotein, making it easier for P-gp substrate drugs to be absorbed and affecting their distribution in the body, which can lead to toxicity. Furthermore, berberine’s ability to lower glucose levels can interact with diabetes medications like insulin, sulfonylureas, or metformin, greatly increasing the risk of hypoglycemia [124]. Berberine is contraindicated during pregnancy and breastfeeding due to its ability to cross the placenta and displace bilirubin from albumin, posing a risk of kernicterus in newborns, and because it is excreted in breast milk [125].

### 6.2. Future Research and Applications

For berberine to become a widely accepted therapeutic agent, its pharmacokinetic limitations must be addressed, and its effectiveness must be thoroughly evaluated across a broader range of conditions.

The development of nanotechnology-based delivery systems is crucial for addressing the clinical limitations of berberine, primarily its low bioavailability due to poor solubility, instability, and susceptibility to efflux. These systems enhance dissolution, protect berberine from degradation, improve absorption (potentially through lymphatic transport), and enable targeted delivery. Effective nanoplatforms include solid lipid nanoparticles, chitosan-based polymeric nanoparticles, liposomes, niosomes, magnetic nanoparticles, silver nanoparticles, and lyotropic liquid crystalline nanoparticles. According to Iqbal et al. [126], the use of these nanocarriers enhances berberine’s uptake by cancer cells and its therapeutic effect against malignancies, including breast, liver, nasopharyngeal, and prostate cancer. Key advantages comprise controlled release kinetics and the potential for synergistic co-administration with agents such as doxorubicin or silver nanoparticles to overcome multidrug resistance (MDR).

There are also various conventional formulations, such as pH-dependent tablets, floating pellets or beads, solid dispersions, and topical hydrogels, developed to enhance dissolution, provide gastric retention, target specific sites (e.g., the colon), or improve permeation, thereby significantly increasing bioavailability and enabling sustained release [127]. While conventional systems offer easier processing and scalability, polymeric systems provide greater control and improved bioavailability but face challenges such as biocompatibility issues, complex manufacturing, scalability problems, and potential toxicity from monomers. Raju et al. [127] also described advanced carriers, including microparticles, microcapsules, microspheres, or dendrimers, that effectively encapsulate berberine, achieving high drug loading and dramatically enhancing pharmacokinetic parameters while improving oral absorption.

As an example, encapsulating berberine in Solid Lipid Nanoparticles significantly increased its plasma levels and bioavailability in diabetic (db/db) mice, resulting in greater reductions in fasting blood glucose, body weight gain, and insulin resistance compared to free alkaloid. The nanoparticles predominantly accumulated in the liver, where they effectively reduced hepatic steatosis by modulating fat metabolism genes (such as by increasing CPT-1 and suppressing SREBP-1c), with a 100 mg/kg dose showing markedly stronger effects than an equivalent free berberine dose. The authors confirmed that the enhanced bioavailability and liver targeting led to superior improvements in glucose tolerance, insulin sensitivity, and protection of islet function in diabetic models [128].

The study by Demet et al. [129] demonstrated that liposomal encapsulation enhances the stability and bioavailability of berberine, highlighting its potential, particularly in its liposomal form, to induce browning (via uncoupling protein 1, UCP1) and modulate lipid metabolism in adipocytes, laying the foundation for novel anti-obesity strategies. In their study, berberine was successfully encapsulated in liposomes, which provided sustained release and significantly improved its physical and chemical stability compared to free berberine, especially protecting it from degradation under light. Both free and liposomal berberine increased the expression of the key browning marker UCP1 in 3T3-L1 adipocytes during both differentiation and maturation processes. Moreover, liposomal berberine (20 µM) significantly reduced triglyceride levels during the differentiation treatment, indicating a potential role in modulating lipid metabolism in early adipocyte development [129].

Researchers have also attempted to enhance berberine’s bioavailability by creating numerous derivatives. Key strategies included chemical modifications (e.g., 9-*O*-esters, 12-aryl substitutions, carbohydrate conjugates, and hybrids such as metformin-berberine) to enhance absorption and reduce intestinal efflux. The modified compounds demonstrated significantly stronger effects in tests, including enhanced hypoglycemic effects (via the AMPK/PI3K/Akt pathways), lipid-lowering capabilities, anti-inflammatory actions, and cytotoxicity against cancer cells compared to natural berberine. Moreover, studies have confirmed the efficacy of these derivatives using in vitro assays (e.g., glucose consumption in HepG2 cells and insulin sensitivity in adipocytes) and in vivo diabetic and obese animal models [130].

Although berberine exhibits promising therapeutic effects in obesity and metabolic disorders, several critical gaps remain to be addressed for successful clinical translation. Regulatory hurdles stem from variability in compound purity, inconsistencies in commercial formulations, and the absence of standardized clinical protocols guiding berberine usage. Furthermore, much of the current evidence originates from preclinical studies or small-scale clinical trials, necessitating larger, well-controlled human studies to establish robust safety and efficacy profiles. Future research should prioritize longitudinal human investigations into berberine’s effects on the gut microbiome and its metabolic consequences, as well as standardized dosing strategies to optimize therapeutic outcomes. Additionally, human-based multi-omics trials are essential to confirm molecular targets and mechanistic pathways identified in preclinical research, paving the way for precision medicine applications. Addressing these challenges will be pivotal for integrating berberine into evidence-based clinical practice as an adjunctive therapy in obesity and metabolic health management.

## 7. Conclusions

Berberine demonstrates substantial anti-obesity and metabolic regulatory potential through its multifaceted actions on key biological pathways. By activating AMPK, it promotes lipid oxidation, mitochondrial biogenesis, and glucose homeostasis, while modulation of the gut microbiota contributes to improved metabolic signaling via increased SCFA production and reduced systemic inflammation. Berberine also influences adipokine profiles, favoring insulin sensitivity and reduced inflammatory responses. Insights from metabolomics have expanded our understanding of berberine’s systemic effects, highlighting its roles in bile acid modulation, carnitine-mediated fatty acid transport, and TCA cycle optimization, collectively contributing to reduced adipogenesis and ectopic lipid deposition. However, its clinical application faces significant challenges, including poor oral bioavailability, gastrointestinal side effects, and potential drug interactions related to CYP450 and P-glycoprotein substrates. Innovative delivery systems such as lipid nanoparticles, cyclodextrin complexes, and structurally modified derivatives, including berberine–metformin hybrids, are under investigation to overcome these barriers and enhance therapeutic efficacy. Future research integrating multi-omics approaches in human studies is crucial to validate mechanistic insights and guide personalized therapeutic strategies. With these advancements, berberine holds promise as a complementary, evidence-based option in the management of obesity and metabolic disorders.

## Figures and Tables

**Table 1:** Key physicochemical properties of berberine chloride hydrate.

Property	Value/Description
Molecular formula	C_20_H_17_NO_4_HCl·2H_2_O
Molecular weight	407.65 g/mol
Appearance	Yellow crystalline solid
Melting point	145.1–146.7 °C
Solubility	pH-dependent: 9.69 mM (pH 7.0, 37 °C); insoluble in nonpolar solvents [22]
Stability	<5% degradation (6 months, pH 1.2–9.0, 40 °C) [21]
Fluorescence	Absorption: 350 nm; emission: 530 nm [19]

**Table 2:** Distribution of berberine in diverse plant taxa. A compilation of research findings from 2024 to 2025.

Plant Taxa	Plant Family	Plant Part Used	Geographical Origin	Concentration (Reported Value)	Analytical Method for Quantification	Extraction Method	Reference
*Phellodendron amurense* Rupr.	Rutaceae	Bark	Dujiangyan, Sichuan, China	0.243 mg/g of extract	UV-Vis spectrophotometry (λmax = 354 nm); UPLC-Q-TOF-MS/MS	Microwave-assisted extraction (ethanol solvent; 350 W; 75 s; material–liquid ratio 1:25 g/mL)	[23]
*Tinospora cordifolia* (Willd.) Miers	Menispermaceae	Stems	Kolkata, West Bengal, India	Not reported	HPLC-DAD	hydro–alcoholic (ethanol–water, 60:40)	[24]
*T. cordifolia*	Menispermaceae	Stems	Kharagpur, West Bengal, India	Not reported	LC-MS/MS	UAE (22.5:1 solvent-to-solid ratio, 40 min sonication time, 75% ethanol)	[25]
*Berberis darwinii* Hook.	Berberidaceae	Roots	Temuco, Chile	Up to 26,482.20 µg/g dry weight; mean: ~1437–4427 µg/g across locations	HPLC-DAD	Freeze-drying of plant tissues, acid extraction, liquid–liquid extraction with chloroform	[26]
*B. darwinii*		Stems	Temuco, Chile	Up to 6639.58 µg/g dry weight; mean: ~828–2035 µg/g across locations			
*B. darwinii*		Seeds	Valdivia, Chile	Up to 1181.75 µg/g dry weight; mean: ~89–467 µg/g across locations			
*B. darwinii*		Leaves	Nueva Imperial, Chile	Up to 511.02 µg/g dry weight; mean: ~24–118 µg/g across locations			
*Coptis chinensis* Franch.	Ranunculaceae	Rhizomes	Hue, Vietnam	15.2–46.2 mg/g dry weight	HPLC	UAE (solvents: 96% lactic acid, 40% malic acid, or 88% pyruvic acid (*w*/*w*), liquid–solid ratio: 30 mL/g, temperature: 60 °C (lactic acid), 80 °C (malic acid), 75 °C (pyruvic acid), time: 30 min	[27]
*Mahonia bealei* (Fort.) Carr.	Berberidaceae	Leaves	Sichuan Province, China	Not reported	UPLC-Q-TOF-MS/MS	Ultrasonic synergistic high-speed homogeneous extraction (50% ethanol, 1:10 solid–liquid ratio, 20,000 r/min homogenization + 300 W ultrasonication for 30 min)	[28]
*C. chinensis* Franch. (as a part of a multi-herb formula)	Ranunculaceae	Not specified	China	Not reported	HPLC (UV detector)	Reflux with 75% ethanol (1:8 ratio)	[29]
*T. cordifolia*	Menispermaceae	Stems	Not specified	2.49% (*w*/*w*)	HPTLC	MAE (500 W, 3 min, 1:15 ratio, 50 °C, methanol)	[30]
*B. aristata* DC.	Berberidaceae	Not specified	Not specified	Not specified	UHPLC-DAD	Ultrasonic bath (10 min, 25 °C, methanol)	[31]
*C. teeta* Wall.	Ranunculaceae	Rhizomes	Arunachal Pradesh, India	212.18 ppm for MAE; 162.96 ppm for UAE	HPLC (UV detector)	MAE (65% solvent concentration, 310 W power, 30 min extraction time, and 1:39 g/mL solid–liquid ratio); UAE (36% solvent concentration, 160 W ultrasound power, 10 min extraction time, and 1:78 g/mL solid–liquid ratio)	[32]
*Helicteres isora* L.	Malvaceae	Fruits	Neyshabur, Iran	21.37% of the total area	GC-MS	Subcritical water extraction (175 °C, 4 mL/min, 120 min, 4 g sample)	[33]
*Thalictrum foliolosum* DC.	Ranunculaceae	Roots	Almora district, Uttarakhand, India	13.14 mg/g dry weight	HPLC-PDA	UAE using NADES (tartaric acid–glycerol, 1:1 molar ratio with 30% water content, duty cycle 80%, liquid-to-solid ratio of 30 mL/g, time: 14 min)	[34]
*B. vulgaris*	Berberidaceae	Roots	Poland	111.06 mg/g	HPTLC	70% aqueous ethanol, sonicated (2 cycles of 20 min each, 700 W, 50 °C)	[35]
*Phellodendron amurense* Rupr.	Rutaceae	Bark	Thua Thien Hue, Vietnam	50.88 mg/g	HPLC	UAE (46% aqueous malic acid, 80 °C, 33.5 min, 26.8 mL/g solvent-to-solid ratio)	[36]
*Cayratia albifolia* C.L.Li	Vitaceae	Roots	Hunan Province, China	Not specified	LC-MS/MS	Boiled in 95% ethanol for 1 h	[37]
*B. koreana* Palib.	Berberidaceae	Twigs	Stobierna, Poland	0.042–0.067%	HPTLC	Ultrasonic bath (70% ethanol, 50 °C, 2 × 20 min)	[38]
*Berberis* × *ottawensis* “Superba”				0.103%			
*B. thunbergii* DC.				0.364–0.676%			
*C. chinensis* Franch.	Ranunculaceae	Roots	China	Up to 10%	HPLC	DES-based UAE (choline chloride–urea (1:2 molar ratio) in 50% aqueous DES, 150 W, 60 °C, 15 min)	[39]
*C. chinensis*	Ranunculaceae	Rhizomes	Sichuan, China	39.57–77.12 mg/g dry weight	UHPLC-PDA	DES-UA-MSPD (betaine–acrylic acid (1:4) with 50% water, silica gel as a sorbent in a 1:1 ratio with the sample, 1:62 g/mL solid-to-DES ratio, 200 W of ultrasonication for 6 min)	[40]
*B. vulgaris* L.	Berberidaceae	Stem bark	Oratia, Buzau County, Romania	78.95 µg/g dry extract	HPLC-DAD	Hydro–ethanolic (50% ethanol) reflux extraction	[41]
*B. vulgaris* L.	Berberidaceae	Roots	Poland (commercial)	Not reported	HPLC-ESI-Q-TOF-MS/MS	ASE with methanol (90 °C, 96 bar, 4 cycles)	[42]
*B. vulgaris* L.	Berberidaceae	Stems	Tbilisi, Georgia				
*Coscinium fenestratum* (Gaertn.) Colebr.	Menispermaceae	Stems and Roots	Thua Thien Hue, Vietnam	38.23 mg/g	HPLC	60% lactic acid (*w*/*w*), liquid–solid ratio of 17.25 mL/g, 66 °C, 20 min	[43]
*Annickia affinis* (Exell) Versteegh & Sosef	Annonaceae	Stem bark	Ovia area, Benin City, Edo State, Nigeria	~0.02% *w*/*w*	LC-ESI-MS/MS	Direct methanol extraction (pulverized stem bark shaken in methanol, 120 rpm, 72 h, 25 °C)	[44]
*Thalictrum* spp. (57 batches)	Ranunculaceae	Stems and roots	Yunnan and Xizang Provinces, China	0.01–12.44 mg/g	LC-MS/MS	75% methanol, solid-to-liquid ratio of 1:100, 60 min of sonication	[45]

MAE—microwave-assisted extraction; UPLC-Q-TOF-MS/MS—ultra-performance liquid chromatography–quadrupole time-of-flight tandem mass spectrometry; UAE—ultrasound-assisted extraction; HPLC-DAD—high-performance liquid chromatography with a diode array detector; LC-MS/MS—liquid chromatography–tandem mass spectrometry; HPTLC—high-performance thin-layer chromatography; GC-MS—gas chromatography–mass spectrometry; PDA—photo diode array; NADES—natural deep eutectic solvent; DES-UA-MSPD—deep eutectic solvent ultrasound-assisted matrix solid-phase dispersion; HPLC-ESI-Q-TOF-MS/MS—high-performance liquid chromatography–electrospray ionization–quadrupole time-of-flight tandem mass spectrometry; ASE—accelerated solvent extraction.

## Data Availability

No new data were created or analyzed in this study. Data sharing is not applicable to this article.

## Abbreviations

The following abbreviations are used in this manuscript:

Akt	Protein kinase B
AMP	Adenosine monophosphate
AMPK	AMP-activated protein kinase
AOM	Anti-obesity medication
ASE	Accelerated solvent extraction
ATP	Adenosine triphosphate
BA	Bile acid
BBR	Berberine
BCAA	Branched-chain amino acid
BSEP	Bile salt export pump
C/EBP-α	CCAAT/enhancer-binding protein alpha
CI	Color Index
CPT1A	Carnitine palmitoyltransferase 1A
CRP	C-reactive protein
CVD	Cardiovascular disease
CYP2C9	Cytochrome P450 2C9 enzyme
CYP2D6	Cytochrome P450 2D6 enzyme
CYP3A4	Cytochrome P450 3A4 enzyme
CYP7A1	Cholesterol 7α-hydroxylase
DES-UA-MSPD	Deep eutectic solvent ultrasound-assisted matrix solid-phase dispersion
ERK	Extracellular signal-regulated kinase (subgroup of MAPK)
FASN	Fatty acid synthase
FXR	Farnesoid X receptor
G6Pase	Glucose-6-phosphatase
GC-MS	Gas chromatography-mass spectrometry
GIP	Glucose-dependent insulinotropic polypeptide
GLUT4	Glucose transporter type 4
GLP-1	Glucagon-like peptide-1
HbA1c	Glycated hemoglobin
HFD	High-fat diet
HO-1	Heme oxygenase-1
HPβCD	2-hydroxypropyl-β-cyclodextrin
HPLC-DAD	High-performance liquid chromatography with a diode array detector
HPLC-ESI-Q-TOF-MS/MS	High-performance liquid chromatography–electrospray ionization–quadrupole time-of-flight tandem mass spectrometry
HPTLC	High-performance thin-layer chromatography
IBD	Inflammatory bowel disease
IKK-β	Inhibitor of nuclear factor kappa-B kinase subunit beta
IL-1β	Interleukin-1 beta
IL-6	Interleukin-6
IRS-1	Insulin receptor substrate 1
JNK	c-Jun N-terminal kinase (subgroup of MAPK)
LC-MS/MS	Liquid chromatography–tandem mass spectrometry
LDL	Low-density lipoprotein
LDL-C	Low-density lipoprotein cholesterol
LDLR	Low-density lipoprotein receptor
LPS	Lipopolysaccharide
LXRα	Liver X receptor alpha
MAE	Microwave-assisted extraction
MAPK	Mitogen-activated protein kinase
MASLD	Metabolic dysfunction-associated steatotic liver disease
MASH	Metabolic dysfunction-associated steatohepatitis (inflammatory form of MASLD)
MCP-1	Monocyte chemoattractant protein-1
MDR	Multidrug resistance
mTOR	Mechanistic target of rapamycin
MW	Molecular weight
NADES	Natural deep eutectic solvent
NAFLD	Non-alcoholic fatty liver disease
NCD	Non-communicable disease
NF-κB	Nuclear factor kappa-light-chain-enhancer of activated B cells
NHANES	National Health and Nutrition Examination Survey
NO	Nitric oxide
NRF-1	Nuclear respiratory factor 1
Nrf2	Nuclear factor erythroid 2-related factor 2
Opa1	Optic atrophy 1 (mitochondrial fusion protein)
OTU	Operational taxonomic unit
p38	p38 mitogen-activated protein kinase (subgroup of MAPK)
P-gp	P-glycoprotein
PDA	Photo diode array
PEPCK	Phosphoenolpyruvate carboxykinase
PHB2	Prohibitin 2 (mitophagy receptor)
PI3K	Phosphatidylinositol 3-kinase
PGC-1α	Peroxisome proliferator-activated receptor gamma coactivator 1-alpha
PINK1	PTEN induced kinase 1 (mitophagy regulator)
PPAR-γ	Peroxisome proliferator-activated receptor gamma
PPAR-δ	Peroxisome proliferator-activated receptor delta
PYY	Peptide YY
ROS	Reactive oxygen species
SCFA	Short-chain fatty acid
SHP	Small heterodimer partner
SIRT1	Sirtuin 1 (NAD+-dependent deacetylase)
SREBP-1c	Sterol regulatory element-binding protein-1c
T1DM	Type 1 diabetes mellitus
T2DM	Type 2 diabetes mellitus
TCA	Tricarboxylic acid cycle (Krebs cycle)
TC	Total cholesterol
TFAM	Mitochondrial transcription factor A
TG	Triglycerides
TGR5	Takeda G protein-coupled receptor 5
TMAO	Trimethylamine N-oxide
TNF-α	Tumor necrosis factor alpha
TORC2	Target of rapamycin complex 2
UAE	Ultrasound-assisted extraction
UCP1	Uncoupling protein 1
UPLC-Q-TOF-MS/MS	Ultra-performance liquid chromatography–quadrupole time-of-flight tandem mass spectrometry
WHO	World Health Organization
ZO-1	Zonula occludens-1 (tight-junction protein)
